# Knocking on Heaven's Door: Are Novel Invaders Necessarily Facing Naïve Native Species on Islands?

**DOI:** 10.1371/journal.pone.0151545

**Published:** 2016-03-15

**Authors:** Agathe Gérard, Hervé Jourdan, Alexandre Millon, Eric Vidal

**Affiliations:** 1 Institut Méditerranéen de Biodiversité et d’Ecologie marine et continentale (IMBE), Aix-Marseille Université, UMR CNRS—IRD–UAPV, UMR 237 IRD, Centre IRD Nouméa—BP A5, 98848, Nouméa Cedex, Nouvelle-Calédonie; 2 Institut Méditerranéen de Biodiversité et d’Écologie marine et continentale (IMBE), Aix-Marseille Université, UMR CNRS—IRD—UAPV, Europôle de l’Arbois, BP 80, 13545, Aix-en-Provence, France; Muséum national d'Histoire naturelle, FRANCE

## Abstract

The impact of alien predator species on insular native biota has often been attributed to island prey naïveté (i.e. lack of, or inefficient, anti-predator behavior). Only rarely, however, has the concept of island prey naïveté been tested, and then only a posteriori (i.e. hundreds or thousands of years after alien species introduction). The presence of native or anciently introduced predators or competitors may be crucial for the recognition and development of adaptive behavior toward unknown predators or competitors of the same archetype (i.e. a set of species that occupy a similar ecological niche and show similar morphological and behavioral traits when interacting with other species). Here, we tested whether two squamates endemic to New Caledonia, a skink, *Caledoniscincus austrocaledonicus*, and a gecko, *Bavayia septuiclavis*, recognized and responded to the odor of two major invaders introduced into the Pacific islands, but not yet into New Caledonia. We chose one predator, the small Indian mongoose *Herpestes javanicus* and one competitor, the cane toad *Rhinella marina*, which belong respectively to the same archetype as the following two species already introduced into New Caledonia in the nineteenth century: the feral cat *Felis catus* and the golden bell frog *Litoria aurea*. Our experiment reveals that geckos are naïve with respect to the odors of both an unknown predator and an unknown competitor, as well as to the odors of a predator and a competitor they have lived with for centuries. In contrast, skinks seem to have lost some naïveté regarding the odor of a predator they have lived with for centuries and seem “predisposed” to avoid the odor of an unknown potential competitor. These results indicate that insular species living in contact with invasive alien species for centuries may be, although not systematically, predisposed toward developing adaptive behavior with respect to species belonging to the same archetype and introduced into their native range.

## Introduction

Alien predators are known to inflict greater damage on prey populations than do native predators, especially on islands [1–3]. Invasive alien species are thus the most significant drivers of population declines and species extinctions on island ecosystems worldwide [4,5]. Naïveté, defined as a lack of predator recognition and of effective anti-predator behavior owing to the lack of a common evolutionary history with a given predator, is considered as particularly characteristic of island native species [6,7]. Diamond and Case [8] were among the first to suggest that the devastating impacts of invasive alien predators on their new environment might be due to the naïveté of island species.

However, naïveté is not restricted to predation and can be observed in connection with any antagonistic interactions. The inability to recognize and respond effectively to a novel competitor, defined as “competitive naïveté”, could also potentially affect access to resources and hence population growth and survival [9,10]. Avoiding areas with introduced predators or competitor odor, which reduces the likelihood of costly or lethal encounters, may be an important adaptive trait in native species. The few studies on recognition and avoidance of the odor of introduced species have investigated changes in ventilatory frequency, choice of foraging sites or trapping success of native species in presence of odors, hundreds or thousands of years after alien species introduction [9,11–16].

On the whole, studies on island species naïveté have examined how native species respond to introduced predators or competitors with which they have co-existed for the centuries or millennia since their introduction, allowing the native species time to acquire “eco-evolutionary experience”[17]. The concept of “eco-evolutionary experience” posits that biotic interactions maintained during the evolutionary history of species influences the outcome of present-day interactions between native and introduced species [17]. Experience is defined here as familiarity not with particular species, but rather with archetypes of interaction partners [18]. An archetype refers to a set of species that occupy a similar ecological niche and show similar morphological and behavioral traits when interacting with other species [7]. The presence of such an archetype could influence the naïveté level of insular prey species toward a novel species of the same archetype [6,7]. According to the “common constituent hypothesis”, carnivorous predators share a nonspecific common odor that could be perceived as a danger signal, thereby inducing generalized avoidance even in naïve species [19,20]. Therefore, the presence of native or anciently introduced predators or competitors may be crucial for the recognition and development of adaptive behavior toward unknown species of the same archetype [17,19–22].

To the best of our knowledge, however, the naïveté of island native species with respect to the odor of predators or competitors not yet introduced into their natural or dispersal range has never been explored. Currently, the spread of exotic species is accelerating under the influence of a number of global change drivers such as world trade, global transport, land-use and climate change [23–25], and various species (including alien predators or competitors) are likely to be introduced into new regions. Biodiversity hotspots, especially islands or groups of islands, will be disproportionately exposed to a high number of invasive alien species, both currently and in the future [26].

**Fig 1:**
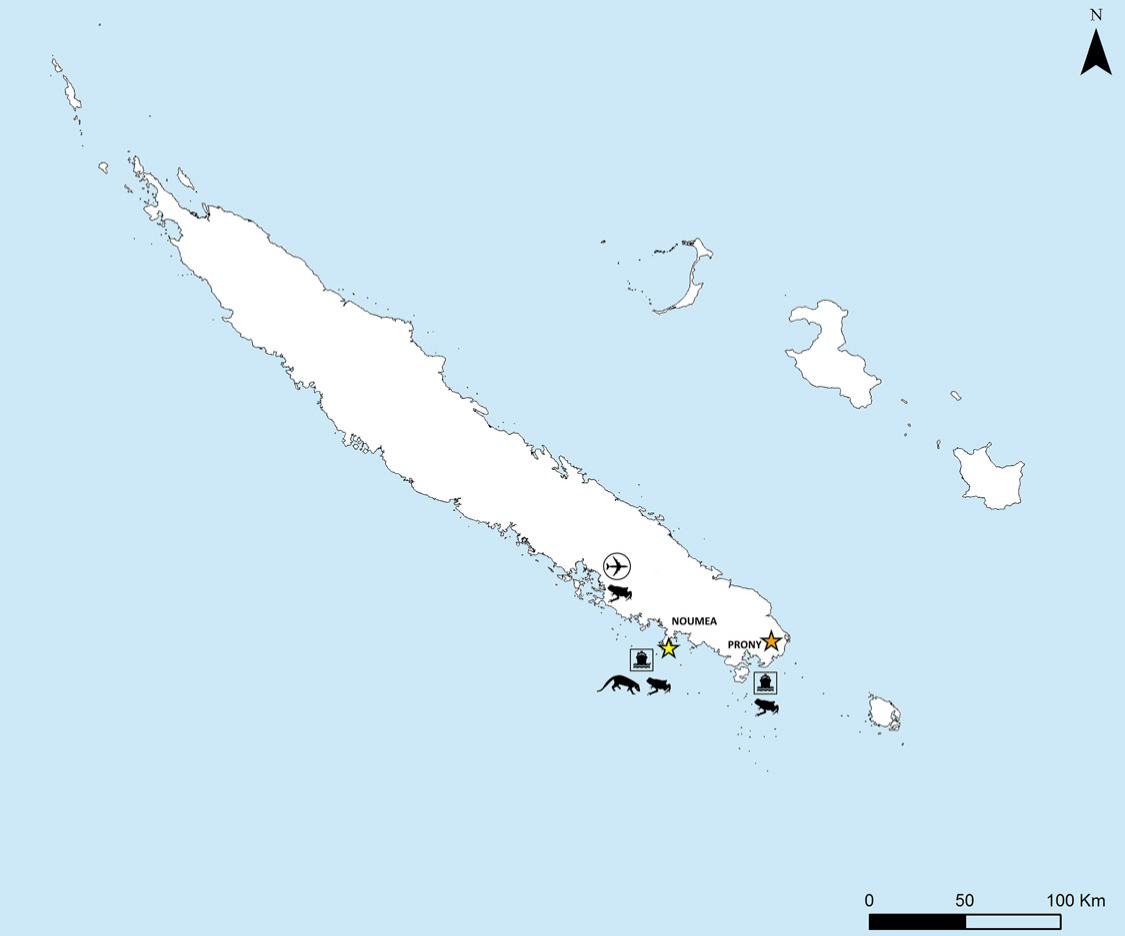
Map of New Caledonia with interception points (international airport; Noumea harbor and Prony industrial site) for the small Indian mongoose (*Herpestes javanicus*) and the cane toad (*Rhinella marina*) and capture sites for the common litter skink (*Caledoniscincus austrocaledonicus*) (yellow star) and the pale striped Bavayia gecko (*Bavayia septuiclavis*) (orange star).

**Fig 2:**
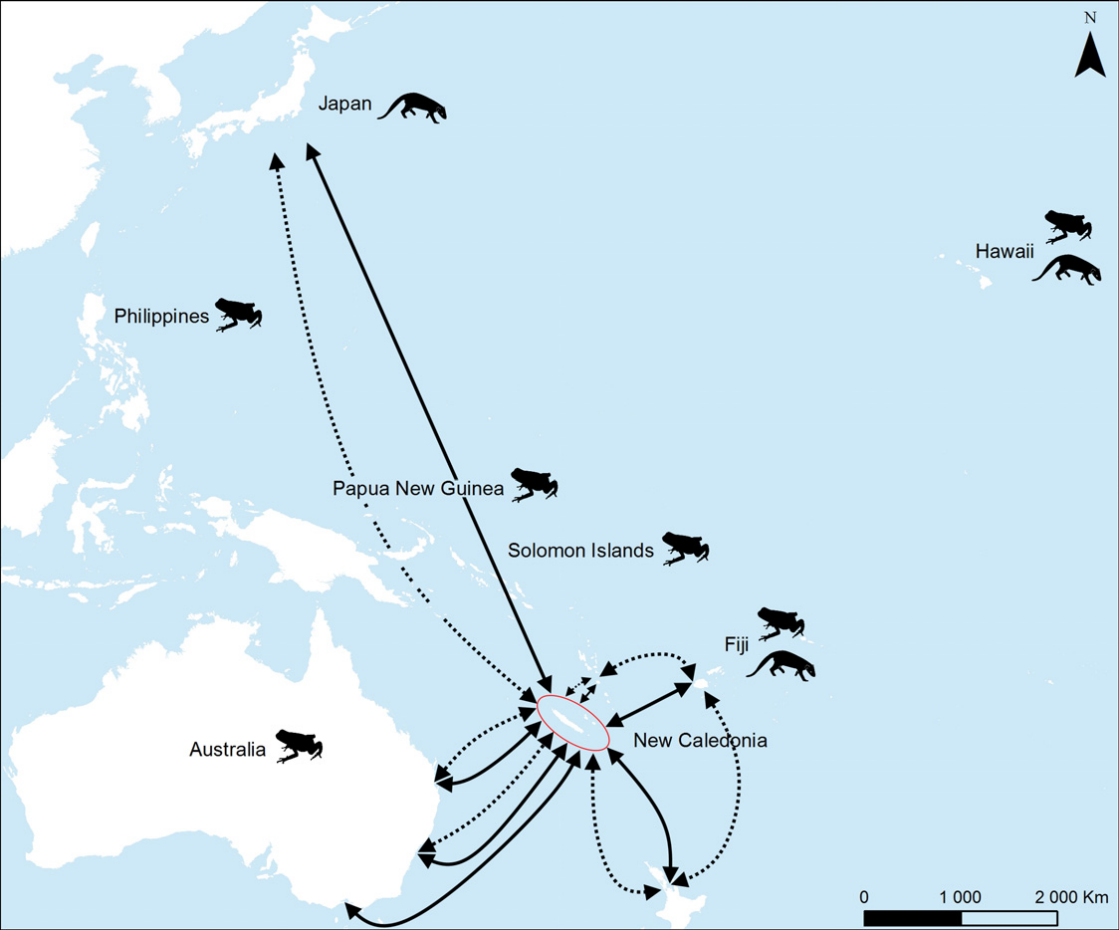
Map of the Pacific context regarding invasive threat by mongoose and cane toad for New Caledonia. Islands invaded by the small Indian mongoose (*Herpestes javanicus*). Islands invaded by the cane toad (*Rhinella marina*). Main air (solid arrow) and maritime shipping links (dotted arrow) for New Caledonia (red ellipse).

Thus, the Pacific region contains eight insular hotspots [27] whose exceptional biodiversity is severely threatened by biological invasions [26]. For instance, the small Indian mongoose (*Herpestes javanicus*) (Hodgson 1836) and the cane toad (*Rhinella marina*) (Linné, 1758) are two of the most devastating animal invaders in the Pacific islands, where they have been accidentally or intentionally introduced [28–30]. The mongoose and the cane toad have severe deleterious impacts on many native species, including endemic squamates [28,31,32]. Their direct (i.e. predation) and indirect (i.e. competition for food or shelter sites) impacts induce reductions in abundance or the extirpation of squamate species [28,33–36]. Squamates are commonly considered to have limited cognitive abilities of memory and learning [37] and for this reason may be predisposed to a high level of naïveté. However, studies have shown that some squamate species are able to detect and avoid the odor of native or already introduced predators [16,38–41]. New Caledonia is one of the major biodiversity hotspots in the Pacific, especially for terrestrial squamates with high diversity (105 described species and probably 20–30 new ones currently under description) and extremely high levels of narrow-range endemism (92.8%) [42,43]. The small Indian mongoose and the cane toad have already been intercepted at three points of entry to New Caledonia [44–47] (Fig 1), due to the many air and maritime links between New Caledonia and several Pacific countries already invaded by these two species (Fig 2). Their future introduction and establishment therefore represents a significant ascertained threat to the New Caledonian fauna, including herpetofauna.

In this study, we tested whether two endemic squamates of New Caledonia, a diurnal skink (*Caledoniscincus austrocaledonicus*) and a nocturnal gecko (*Bavayia septuiclavis*), recognize and respond to the odor of these two potential invaders (hereafter termed “unknown” species), and to the odor of two species already introduced into New Caledonia (hereafter termed “invasive alien” species), and belonging respectively to the same predator and competitor archetypes (the feral cat *Felis catus* and the golden bell frog *Litoria aurea*). We considered the mongoose as an archetype of the mammal predator, and the cane toad as an archetype of the competitor. We assumed that there would be exploitative competition for resources and interference competition with a risk of predation for retreat sites, due to overlapping shelter habitat preferences (especially for squamate resting sites or egg laying sites that could be used as resting sites for anurans). We hypothesized that the occurrence of the feral cat and the golden bell frog might influence the responses of naïve species to the two unknown species belonging to the same archetypes.

## Methods

### Ethics statement

*C*. *austrocaledonicus* is neither an endangered nor a protected species according to New-Caledonian or French law. *C*. *austrocaledonicus* were collected on private land belonging to IRD Nouméa. This land is owned by the authors’ institution and therefore no specific permission was required. *B*. *septuiclavis* is a protected species according to the environmental code of Province Sud in New Caledonia, but handling of this species is allowed with specific permission. For this study, *B*. *septuiclavis* were collected at the natural reserve of Pic du Grand Kaori with the permission of Province Sud Environment Office (DENV, decree 2155-2012/ARR/DENV) (Table 1). All of the individuals were temporarily kept in captivity during the experiments with food and water ad libitum before being released in the wild at their initial capture sites. Sampling procedures were specifically approved under the field permit. No specific permission was required to collect faeces, to capture, to temporarily keep in captivity and to release at their initial capture sites the invasive vertebrate species used in this study (Table 1).

**Table 1:** Geographical coordinates of collection sites.

Country	Locality	Latitude	Longitude	Collection
Fiji	Forest Staff village, Colo-I-Suva Forest Park, Suva (privately owned)	18°2'43.15"S	178°23'55.57"E	Faeces of mongoose; Cane toad (n = 3)
Fiji	University of the South Pacific campus, Suva (privately owned)	18°8’59.83"S	178°26’38.48"E	Faeces of mongoose; Cane toad (n = 5)
Hawaii (O'ahu)	University of Hawaii campus, Mānoa (privately owned)	21°17'54.14"N	157°49'5.22"O	Cane toad (n = 17)
Hawaii (O'ahu)	Vacant land near the University of Hawaii campus, Mānoa (publicly owned)	21°17'42.31"N	157°48'50.75"O	Faeces of mongoose
New Caledonia (Main island)	Natural reserve of Pic du grand Kaori (privately owned)	22°16'51.69"S	166°53'46.19"E	*B*. *septuiclavis*
New Caledonia (Main island)	IRD, Noumea (privately owned)	22°18'4.47"S	166°26'38.28"E	*C*. *austrocaledonicus*

### Study species

The native squamates chosen for this experiment are two common species in New Caledonia, the common litter skink (*C*. *austrocaledonicus*) and the pale-striped Bavayia gecko (*B*. *septuiclavis*). They were collected in the field (the skinks from open habitats and the geckos from humid forest) from two areas (Fig 1) currently invaded by an invasive alien predator, the feral cat and an invasive alien competitor, the golden bell frog. Recent diet studies conducted in New Caledonia (unpublished data) show that skinks and geckos are common prey items in the feral cat diet. The golden bell frog is a competitor for resources and shelter for both skinks and geckos, known to sometimes prey upon *C*. *austrocaledonicus* [48].

While it is likely that individuals of both sexes were included in the two squamate species tested, external morphological criteria do not allow reliable distinction of one from the other. Recognition and response by these two squamate species were tested through a retreat site-choice experiment. Retreat site choice provides a robust measure of predator avoidance and has been successfully used in previous studies [16,38–40] to assess lizard avoidance behavior with respect to the odor of native or introduced predators.

The odors were obtained from samples (fresh faeces) collected in the field for both mammal predators, and were obtained from living individuals collected in the field for anurans. Fresh faeces were obtained from Fiji and Hawaii for the mongoose and from southern New Caledonia for the feral cat. Faeces of each species were kept frozen and then thawed and crushed on small pieces of paper towel just before the tests. Competitor odors were obtained from individuals collected in the field. The cane toad odors were obtained from 25 individuals captured in Fiji (n = 8) and Hawaii (n = 17). The golden bell frog odors were obtained from 15 individuals captured in New Caledonia. Cane toad and golden bell frog odors were obtained by placing paper towels on the floor of boxes where 4 or 5 individuals were kept for several days, to collect urine and faeces. In the case of the cane toad, toxic exudates, extracted by pressing the parotid glands of individuals, were also deposited on the soiled paper towels from the boxes. These paper towels were then cut into small pieces of the same size as those carrying the crushed mongoose and feral cat faeces. To distinguish whether responses to odor were specific to predator or competitor odor, or simply responses to any odor, we used as control scent a biological odor outside the experience of the species and individuals tested. We chose the odor of a seabird (Wedge-tailed shearwater, *Puffinus pacificus*), an odor that was not from a predator or competitor and unfamiliar [16]. As this seabird nests exclusively on the coastline (sandy beaches), there is no way that the squamate populations sampled here could have come into contact with it. The control scent odor was obtained by placing paper towels on one fresh corpse of *P*. *pacificus*.

### Retreat site choice experiment

**Fig 3:**
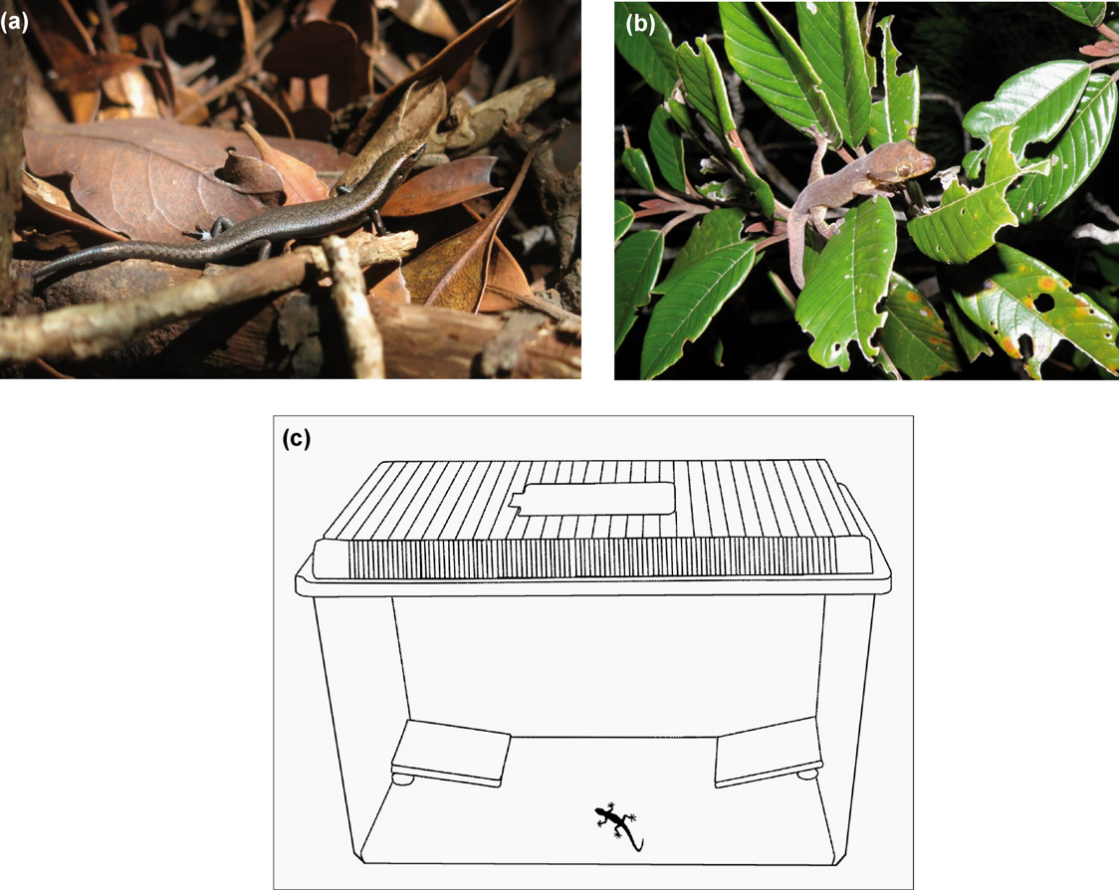
(a) *Caledoniscincus austrocaledonicus* (IMBE) (b) *Bavayia septuiclavis* (A. Gérard) (c) Experimental design: Skinks or geckos are individually placed at the center of the front part of the box at the beginning of the experiment (A. Gérard; GNU Image Manipulation Program 2.8).

Experimental tests were conducted after sunset for diurnal skinks and after sunrise for nocturnal geckos, during the normal period of inactivity when retreat sites are sought by these two species. Squamates were placed in opaque plastic boxes (L × l × h: 32 x 21 x 20 cm) containing two ceramic tiles (7 x 7 cm) as retreat sites (Fig 3). A paper towel was placed under each tile, one treated with odors (predator, competitor or control scent), the other with distilled water (odorless control). New pieces of paper towel, either unscented or randomly selected from the scent-carrying papers for each species, were used for each test. Boxes and tiles were washed with 95% alcohol and dried between trials, and all manipulations were performed wearing latex gloves, to avoid human odor. Choice and position of odor (right or left) were randomly determined before each trial [39,40]. Tests were performed for two hours and the result determined according to the tile under which the lizards were hiding at the end of the experiment. Gérard *et al*. 2014 [16] found in previous experiments that the skink response rate (i.e. total number of tile choices (odorless control and scented tiles) divided by total number of tests) decreased during successive tests with different odors, while the gecko response rate remained high. Therefore, to avoid this undesirable non-response effect, we exposed each skink to one odor only, while each gecko was exposed to all the odors successively, in random order [16]. Tests were performed on 330 adult skinks (66 skinks per odor) and 79 adult geckos. For skinks, each individual was captured several hours before the experiment and placed in a small plastic box with a few leaves and water ad libitum before the beginning of the experiment. For geckos, each individual was maintained in a small plastic box with a few leaves, a piece of wood, crickets, and water ad libitum before and between experiments. Before our experiments, skinks and geckos were acclimated to captivity conditions for 24 hours. At the end of the experiment, all the skinks and geckos were released at their capture sites. Responses were measured by comparing avoidance ratings (number of odorless control tile choices divided by total number of tile choices) between tests with predator/competitor odors and two different controls: no effect of odor (i.e. fixed avoidance rating = 50%) and the control scent (*P*. *pacificus* odor). The non-responding lizards, those that remained outside the tiles and did not choose a retreat site, were excluded from the calculation of the avoidance rating.

### Data analysis

We tested whether skinks and geckos avoided a control scent (i.e., the odor of *P*. *pacificus*) unrelated to any predator/aggressor. Then, we compared lizard avoidance ratings when exposed to predator or aggressor odors, with two different controls: (i) no effect of odor (i.e., random choice corresponding to an avoidance rating of 50%) and (ii) control scent (*P*. *pacificus* odor). These two ways of analyzing the data yield complementary information. The first reveals whether a predator/aggressor odor is attractive/repulsive. The second reveals whether one particular predator/aggressor odor is more attractive/ repulsive than an unknown odor unrelated to any predator/aggressor. Making this comparison should distinguish the strategy “avoid any odor” from the strategy “avoid the odor of a recognized predator”. Statistical analyses were performed using generalized linear models (GLM) for skinks and generalized linear mixed models (GLMM) for geckos, with individual identity as a random factor to control for replicated data coming from a single individual. We also added sequence (order of odor presentation) as a fixed factor in the analysis for geckos. Both models were fitted with a binomial distribution of error (with logit link) and were implemented in R 2.15.0 [49] using the “lme4” library [50].

## Results

**Fig 4:**
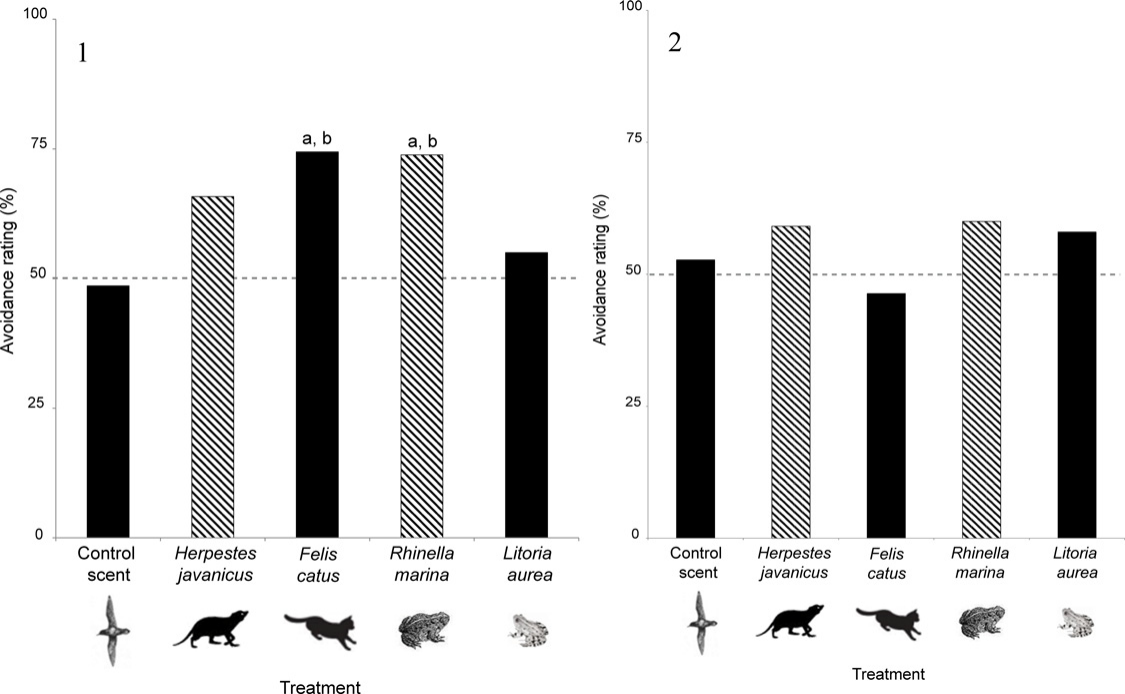
Avoidance rating (number of odorless control tile choices divided by total number of tile choices) of (1) *Caledoniscincus austrocaledonicus* (n = 330) (2) *Bavayia septuiclavis* (n = 79) for choice of retreat sites treated with odors of non-introduced predator (*Herpestes javanicus*), non-introduced (*Rhinella marina*) and extant predator (*Felis catus*), competitor (*Litoria aurea*) or scent control (*Puffinus pacificus*). Black bars denote responses to the odor of species already present in New Caledonia. Hatched bars denote responses to the odor of species not yet introduced into New Caledonia. (a) significant difference with scent control (*P*. *pacificus)*, (b) significant difference with no effect of odor (i.e. fixed avoidance rating = 50%) (dotted line).

We found that neither skinks nor geckos avoided the control scent (*P* = 0.87; *P* = 0.69, respectively) (Fig 4; Table 2) indicating that they are not simply avoiding all scents, but are specifically responding to the scent of predators or competitors. We determined that the odor sequence did not influence gecko retreat site choice (generalized linear mixed models (GLMM; P = 0.12)).

**Table 2:** Results of GLM for (**a**) skinks for tests with predator odors compared with scent control odor (*Puffinus pacificus*) (**b**) skinks for tests with predator odors compared with no effect of odor (i.e. fixed avoidance rating = 50%) (**c**) geckos for tests with predator odors compared with scent control odor (*Puffinus pacificus*) (**d**) geckos for tests with predator odors compared with no effect of odor (i.e. fixed avoidance rating = 50%).

**a**	Estimate	Std. Error	Pr(>|z|)	**c**	Estimate	Std. Error	Pr(>|z|)
Control scent	-0.057	0.338	0.866	Control scent	0.110	0.270	0.686
*H*. *javanicus*	0.711	0.481	0.139	*H*. *javanicus*	0.258	0.409	0.527
*F*. *catus*	1.125	0.486	0.021	*F*. *catus*	-0.256	0.432	0.536
*R*. *marina*	1.093	0.487	0.025	*R*. *marina*	0.296	0.421	0.481
*L*. *aurea*	0.258	0.426	0.545	*L*. *aurea*	0.213	0.394	0.588
**b**	Estimate	Std. Error	Pr(>|z|)	**d**	Estimate	Std. Error	Pr(>|z|)
*H*. *javanicus*	0.654	0.342	0.056	*H*. *javanicus*	0.368	0.307	0.230
*F*. *catus*	1.068	0.350	0.002	*F*. *catus*	-0.147	0.313	0.640
*R*. *marina*	1.036	0.351	0.003	*R*. *marina*	0.405	0.323	0.209
*L*. *aurea*	0.201	0.259	0.439	*L*. *aurea*	0.323	0.286	0.260

Diurnal skinks were more responsive than nocturnal geckos whatever the intercept considered (Fig 4; Table 2). In fact, the skinks avoided two of the odors (the feral cat and the cane toad) (*P*<0.02; Fig 4; Table 2), while the geckos did not avoid any of the odors (*P*>0.21; Fig 4; Table 2), whatever the control considered.

Neither skinks nor geckos avoided the odor of the unknown predator (the small Indian mongoose) (*P*>0.05; Fig 4; Table 2). The odor of the unknown competitor (the cane toad) was avoided by skinks (*P*<0.02; Fig 4; Table 2) but not by geckos (P>0.21; Fig 4; Table 2).

The odor of the feral cat was also avoided by skinks (*P*<0.02; Fig 4; Table 2) but not by geckos (P>0.54; Fig 4; Table 2).

The odor of the golden bell frog was not avoided by skinks (P>0.44; Fig 4; Table 2), nor by geckos (P>0.26; Fig 4; Table 2).

## Discussion

Our experiment suggests that skinks may have lost some naïveté regarding the odor of a predator they have lived with for centuries, and seem “predisposed” to avoid the odor of an unknown potential competitor. In contrast, geckos do not recognize the odors of an unknown predator and an unknown competitor, nor the odors of a predator and a competitor they have lived with for centuries.

According to the prey naïveté hypothesis, the ability of a prey species to detect and avoid novel predators depends on the life history, ecology, and evolutionary history of both predator and prey (e.g. degrees of experience) [6,7,51]. Prey species that have not acquired “eco-evolutionary experience” with predators belonging to certain archetypes are predisposed to high levels of naivety towards novel introduced predators from these archetypes [19]. However, island species that have coexisted with alien predators, for decades or centuries, and developed adaptive avoidance behavior to deal with predation risk, are more likely to avoid unknown predator odor and to associate it with a threat. This is especially true if introduced predators belong the same archetype as the unknown predator, and if risk cues are conserved through phylogeny [6,7,52]. In our study, skinks did not avoid the odor of the small Indian mongoose, an unknown predator with which they do not share either evolutionary or ecological history, whereas they recognized and avoided the odor of feral cats. Thus, although cat and mongoose could be considered as belonging to the same predator archetype (i.e. small carnivorous mammals), the presence of the feral cat for over 150 years in New Caledonia [53] and the recognition and avoidance of its odor by skinks, did not lead to the recognition and avoidance of the small Indian mongoose odor. This result appears to be somewhat inconsistent with the ‘‘common constituent hypothesis”, according to which there is a general nonspecific carnivorous odor that prey are able to assess as a danger signal, even when predators are unfamiliar [19,20]. However, Barrio *et al*. 2010 [54] suggested that in the case of mammals, the common constituent hypothesis may only apply when taxonomic levels are closely related (i.e. between species of the same family). Therefore, due to the age divergence (about 37 million years) between Felidae and Mustelidae, the differences between these two families could be too great for there to be any avoidance of the mongoose odor by skinks [55].

Regarding competitive naïveté toward competitors for food or shelter sites, our study showed that the skink *C*. *austrocaledonicus* avoided the odor of the cane toad despite their lack of shared ecological and evolutionary history. The detection of toxic compounds, present in the exudates from the parotid and skin gland secretion [56] deposited on the paper towels, might explain this avoidance of the odor of the cane toad. This avoidance suggests that skinks could be “predisposed” to recognize and/or to avoid this potential novel competitor and thus potential costly aggressive encounters [57]. Although cane toads and golden bell frogs belong to the same archetype and threat type (anouran), the avoidance of the cane toad odor by skinks does not appear to be linked to the presence in New Caledonia, and at our field sites, of the introduced golden bell frog, since the skinks did not avoid the odor of this frog, despite *ca*.130 years of coexistence [58]. The intensity of a threat is considered important in shaping the development of adaptive behavior [16,40]. Although *L*. *aurea* is known to sometimes prey upon *C*. *austrocaledonicus* [48], these events are probably too sporadic (the *L*. *aurea* diet being mainly composed of insects and tadpoles [59]) to induce pressure that would lead to avoidance of the odor of the golden bell frog by skinks.

In contrast with skinks, the gecko *B*. *septuiclavis*, did not avoid any of the odors of a predator and a competitor they have lived with for centuries, nor the odors of an unknown predator and an unknown competitor. Intensity of threat and differences in the biology of skinks and geckos have been advanced to explain the observed difference in behavior between these two groups of squamates when faced with introduced predators [16]. Skinks are active foragers using their chemical discrimination abilities to detect prey, whereas geckos tend to be ambush foragers relying less on chemical discrimination [60]. Against anuran species, squamate species foraging in arboreal habitats (like geckos) were found to be at lower risk of decline in abundance than squamate species foraging on the ground and in riparian environments in tropical Australia [61]. Moreover, the selection of a retreat site to avoid a risk of predation during the inactive period, i.e. the specific behavior we tested in our experiment, might be more crucial for skinks than for geckos. In fact, diurnal skinks spend most of their time in retreat sites at night, when nocturnal species like the feral cat are most active and when predation risk at retreat sites is therefore highest. Selecting a retreat site in an area without feral cat odors may thus be crucial for skinks, so as to limit predation risk during peak predator activity periods. Conversely, nocturnal geckos occupy retreat sites during the day, when nocturnal species like the feral cat are less active. Consequently, there may be less predation pressure at retreat sites and less need to avoid retreat sites with odors of predators for geckos than for skinks.

To conclude, this study thus shows that island species living in contact with invasive alien species for centuries may be, although not systematically, predisposed toward developing adaptive behavior with respect to species belonging to the same archetypes and introduced into their native range. The probable future establishment of the small Indian mongoose and the cane toad in New Caledonia could therefore have serious consequences on the exceptional species richness of squamates in New Caledonia. The establishment of the small Indian mongoose would add predation pressure on New-Caledonian squamates, some of which have an extremely high level of narrow range endemism [62] and are already severely threatened by feral cats and different species of introduced rodents [63]. The establishment of the cane toad could lead to lethal ingestion by large squamates (e.g. *Lioscincus nigrofasciolatum*, *Phoboscincus* sp.) and could impact the smaller endemic species via predation and competition for habitat or retreat sites [61].

Unfortunately, a number of global change drivers are accelerating the spread of exotic species. Climate change influences all invasive alien species by affecting their spread and colonization of new habitats [23]. The fragmentation of the landscape by land use intensification reduces the ability of resident species to resist invasion [23]. The growth and development of world markets [25], the adoption of novel species as pets [64], are factors that facilitate or create conditions favorable to the arrival or progression of certain species. It would be interesting to realize similar studies in various island contexts, for example with unknown predators or competitors of native species, both belonging and not belonging to the same archetype as already introduced species. This could reveal patterns of responses by naïve species and provide additional insights into the factors affecting sensitivity in native species exposed to new predators or competitors.

The increasing velocity of toad and mongoose expansion in areas where they have been introduced [65,66], together with the difficulty or impossibility of creating barriers to further spread and the probable naïveté of local fauna in areas not yet invaded, highlight the importance of promoting biosecurity. New Caledonia, like many other Pacific islands, has become economically- and food-dependent on developed countries [67]. Supplies arrive in New Caledonia mainly by boat and plane, particularly from Fiji and Australia, where these potential major invaders are widely distributed. Therefore, people and supplies coming from these areas need to be the object of particular vigilance during biosecurity controls, to prevent the arrival of two of the most devastating animal invaders in the South Pacific islands. Preventing the introduction of species with a high risk of becoming invasive remains the best way to protect native species and the most cost-effective management strategy [68,69].
